# Supplementary figures and images for: Effect of the online module on leadership in knowledge acquisition among nursing students: A randomized controlled study protocol
Source: PLoS One. 2025 Mar 25;20(3):e0320208. doi: 10.1371/journal.pone.0320208 (PMC11936248; doi:10.1371/journal.pone.0320208)

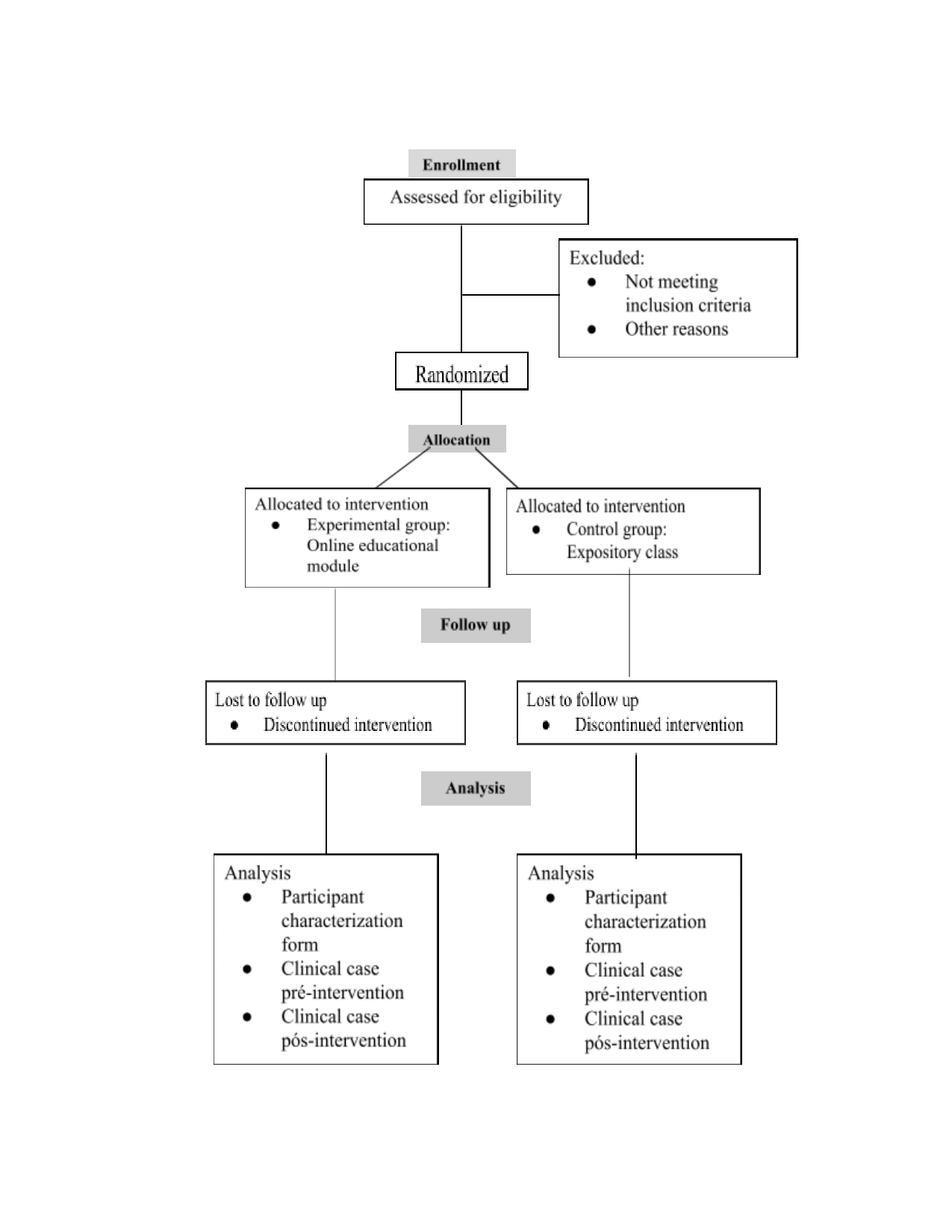

Supplement: S1 Fig 2 — (TIF) [file pone.0320208.s001.tif]

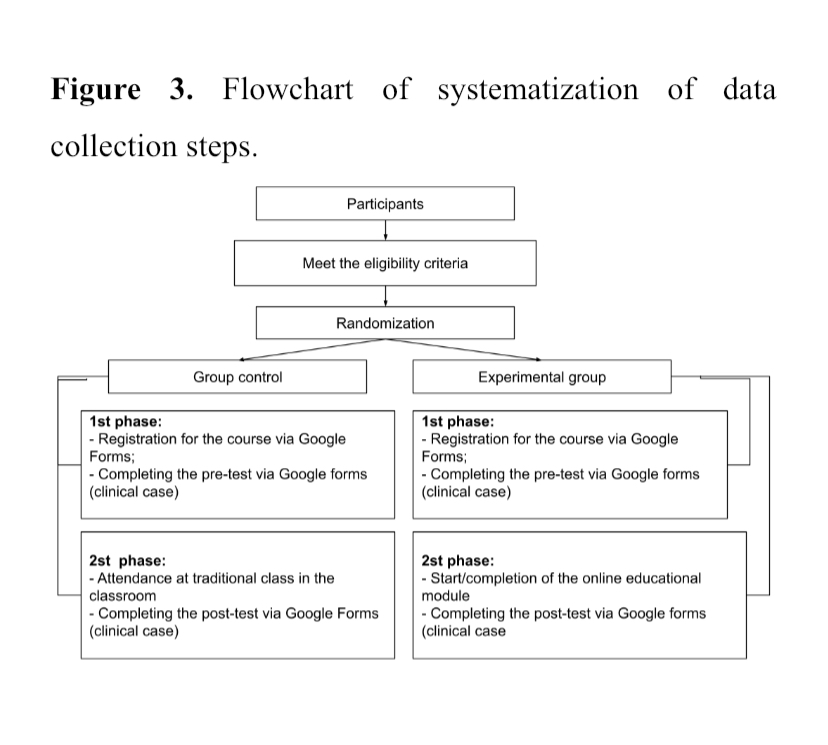

Supplement: S2 Fig 3 — (TIFF) [file pone.0320208.s002.tiff]
