# Supplementary material for: Effect of the online module on leadership in knowledge acquisition among nursing students: A randomized controlled study protocol
Source: PLoS One. 2025 Mar 25;20(3):e0320208. doi: 10.1371/journal.pone.0320208 (PMC11936248; doi:10.1371/journal.pone.0320208)
Supplement: S3 — (DOCX) [file pone.0320208.s003.docx]

**Nursing Student Self-Perception Questionnaire in the Exercise of Leadership**

| Student leader number: _____________________________ | | | | | | | |
| --- | --- | --- | --- | --- | --- | --- | --- |
| **Identification** | | | | | | | |
| Age years)  Gender: ( ) female \| ( ) masculine  Period: ________ | | | | | | | |
| Select the alternative you consider most appropriate  **1. You conceptualize leadership as:**  ( ) The process of exerting influence on people's behavior to achieve objectives in certain situations.  ( ) The process of transforming the behavior of an individual or an organization.  ( ) The legitimate right to exercise power within the organization to obtain obedience from workers.  1.1. ( ) Other concept – specify:  _____________________________________________________________________________________________________________________________________________________________________________________________________  **2. Do you consider yourself a leader?**  ( ) No  2.1 ( ) Yes  Why?  ______________________________________________________________________________________________________________________________________________________________________________________________________  **3. Check the interpersonal skills that you consider necessary for a leader?**  ( ) Communication skills  ( ) Ability to give and receive feedback  ( ) Ability to gain power and exert influence  ( ) All the skills mentioned above | | | | | | | |
| **4. Mark with an**  1. “Never” - I don’t understand the statement.  2. “Rarely” – I eventually notice the statement.  3. “Not always” - I sometimes understand the statement.  4. “Almost always” - I often understand the statement.  5. “Always” - I understand the statement every time.  AT. “Not applicable” - if you are unable to evaluate the issue mentioned. | | | | | | | |
|  |  |  |  |  |  |  |  |
| **Propositions** | | **5** | **4** | **3** | **2** | **1** | **AT** |
| 4.1. I know how to listen to those I lead. | |  |  |  |  |  |  |
| 4.2. I can maintain the interest of those I lead in maintaining and continuing the dialogue . | |  |  |  |  |  |  |
| 4.3. I provide guidance and advice to those I lead, meeting their professional needs. | |  |  |  |  |  |  |
| 4.4. I use verbal communication and pay attention to non-verbal communication in dialogue with those I lead. | |  |  |  |  |  |  |
| 4.5. I contribute to effective communication in working relationships with employees. | |  |  |  |  |  |  |
| 4.6. I give guidance to those I lead and demonstrate how tasks should be carried out , according to their needs. | |  |  |  |  |  |  |
| 4.7. I clarify doubts from employees regarding their tasks . | |  |  |  |  |  |  |
| 4.8. I recognize and value those I lead for what they do or the way they behave . | |  |  |  |  |  |  |
| 4.9. I redirect those I lead by showing a new path to follow when they do not meet expected performance . | |  |  |  |  |  |  |
| 4.10. I periodically monitor the performance of those I lead. | |  |  |  |  |  |  |
| 4.11. I encourage the practice of feedback with those led. | |  |  |  |  |  |  |
| 4.12. I influence those I lead by expanding their skills in favor of effective results. | |  |  |  |  |  |  |
| 4.13. I share decisions with those I lead . | |  |  |  |  |  |  |
| 4.14. I delegate activities to those I lead, sharing responsibilities. | |  |  |  |  |  |  |
| 4.15. I take responsibility for the development of those I lead. | |  |  |  |  |  |  |
| 4.16. I am available to my team members to help them when they are facing any professional difficulties. | |  |  |  |  |  |  |
| 4.17. I ask employees for their opinion on changing a procedure or proposing an operational change. | |  |  |  |  |  |  |
| 4.18. Assistance in defining goals for each leader on my team . | |  |  |  |  |  |  |
| 4.19. I periodically monitor the results presented by each leader. | |  |  |  |  |  |  |
| 4.20. I agree on the necessary deadline for each leader, so that the goals are achieved | |  |  |  |  |  |  |

Source: Adapted from Cardoso, Ramos and D'Innocenzo (2014)
