# Supplementary material for: Effect of the online module on leadership in knowledge acquisition among nursing students: A randomized controlled study protocol
Source: PLoS One. 2025 Mar 25;20(3):e0320208. doi: 10.1371/journal.pone.0320208 (PMC11936248; doi:10.1371/journal.pone.0320208)
Supplement: S5 — (PDF) [file pone.0320208.s005.pdf]

RBR-2dfqmr2 Assessment of the knowledge acquired by nursing students on the topic of Leadership through an Online Module

Date of registration: 02/26/2024 (mm/dd/yyyy)

Last approval date : 02/26/2024 (mm/dd/yyyy)

Study type:

Interventional

Scientific title:

en
Effect of the Online Module on the acquisition of nursing students' knowledge about Leadership: clinical trial

pt-br
Efeito do Módulo Online na aquisição do conhecimento de discentes de enfermagem sobre Liderança: ensaio clínico

es
Effect of the Online Module on the acquisition of nursing students' knowledge about Leadership: clinical trial

Trial identification

- **UTN code:** U1111-1302-2802
- **Public title:**

  en
  Assessment of the knowledge acquired by nursing students on the topic of Leadership through an Online Module

  pt-br
  Avaliação do conhecimento adquirido por estudantes de enfermagem na temática em Liderança por meio de um Módulo Online

- **Scientific acronym:**
- **Public acronym:**
- **Secondaries identifiers:**
  - **75851023.0.0000.5537**
    Issuing authority: Plataforma Brasil
  - **6.599.419**
    Issuing authority: Cômite de Ética em Pesquisa da Universidade Federal do Rio Grande do Norte

Sponsors

- **Primary sponsor:** Departamento de Enfermagem - Universidade Federal do Rio Grande do Norte
- **Secondary sponsor:**
  - **Institution:** Departamento de Enfermagem - Universidade Federal do Rio Grande do Norte
- **Supporting source:**
  - **Institution:** Departamento de Enfermagem - Universidade Federal do Rio Grande do Norte

Health conditions

- **Health conditions:**

en	pt-br
Cognition	Cognição

- **General descriptors for health conditions:**

en	pt-br
**F02.463** Mental Processes	**F02.463** Processos Mentais

- **Specific descriptors:**

en	pt-br
**F02.463.188** Cognition	**F02.463.188** Cognição

Interventions

- **Interventions:**

en	pt-br
An experimental study will be carried out, of the Randomized Clinical Trial type, in which the randomization of participants will be done randomly and the research will be conducted in a single-blind manner, with assessments before and after the intervention. The experimental group will receive intervention through digital educational technology, and the control group will receive a standard intervention consisting of an expository class, both groups will complete a pre-test and post-test. In these scenarios, a pre-test and post-test instrument will be applied, which corresponds to a clinical case. In this research, the Control Group will undergo a traditional lecture lasting four hours and will be placed in a classroom in the Nursing Department of the Federal University of Rio Grande do Norte. While the Experimental Group will undergo an online educational module that lasts four hours as well, which can be consumed over the course of a week, and the intervention will be applied in the Nursing Department's computer laboratory, where participants will receive a login and password to access the platform. With this, we seek to identify the level of learning of students in the control and	Será realizado um estudo experimental, do tipo Ensaio Clínico Randomizado, em que a randomização dos participantes será feita de forma aleatória e a pesquisa será conduzida de maneira mono-cega, com avaliações antes e depois da intervenção. O grupo experimental, contará com a intervenção por meio da tecnologia educacional digital, e grupo controle, receberá uma intervenção padrão composta por aula expositiva, ambos os grupos preencherão um pré-teste e pós-teste. Nestes cenários será aplicado um instrumento de pré-teste e pós-teste, que corresponde a um caso clínico. Nessa pesquisa, o Grupo Controle será submetido a uma aula expositiva tradicional com duração de quatro horas e será disposto em uma sala de aula do Departamento de Enfermagem da Universidade Federal do Rio Grande do Norte. Enquanto o Grupo Experimental será submetido a um módulo educacional online que tem duração de quatro horas também, que poderá ser consumido ao longo de uma semana, e a intervenção será aplicada no laboratório de informática do Departamento de Enfermagem, em que os participantes receberão um login e senha para acessar a plataforma. Com

en	pt-br
experimental groups regarding the teaching of Leadership; and compare the effects of an online educational module with the lecture on the knowledge of Leadership of Nursing students. These comparators will be measured by the improvement in the post-test results of the experimental group in relation to the control group. The target sample size is 60 participants.	isso, busca-se identificar o nível de aprendizagem dos discentes do grupo controle e experimental acerca do ensino de Liderança; e comparar os efeitos de um módulo educacional online com a aula expositiva na conhecimento de Liderança de discentes de Enfermagem. Esses comparadores serão mensurados pela melhoria nos resultados do pós-teste do grupo experimental em relação ao grupo controle. O tamanho da amostra será alvo é 60 participantes.

- **Descriptors:**

en	pt-br
**I02.195** Education, Distance	**I02.195** Aprendizado Online

Recruitment

- **Study status:** Recruiting
- **Countries**
  - Brazil
- **Date first enrollment:** 12/23/2023 (mm/dd/yyyy)
-

Target sample size:	Gender:	Minimum age:	Maximum age:
60	-	18 Y	0

- **Inclusion criteria:**

en	pt-br
People aged 18 or over; both genders; being a student in the seventh period of the Universidade Federal do Rio Grande do Norte - UFRN; Nursing course in the semester of 2024.1; have not consumed content on Leadership and/or have not taken the Health Services Management subject or equivalent subject	Pessoas com idade igual ou superior a 18 anos; de ambos os gêneros; ser discente do sétimo período do curso de Enfermagem da Universidade Federal do Rio Grande do Norte - UFRN no semestre de 2024.1; não ter consumido conteúdo sobre Liderança e/ou não ter cursado a disciplina de Gerências dos Serviços de Saúde ou disciplina equivalente

- **Exclusion criteria:**

en	pt-br
Students in the seventh period; who have already completed other degrees, stricto sensu or lato sensu postgraduate courses, extension courses, free courses, short courses or events that address the topic of leadership	Discentes do sétimo período; que já cursaram outras graduações, pós-graduação stricto sensu ou lato sensu, cursos de extensão, cursos livres, cursos de curta duração ou eventos que abordem a temática de liderança

Study type

- **Study design:**

Expanded access program	Purpose	Intervention assignment	Number of arms	Masking type	Allocation	Study phase
1	Other	Parallel	2	Single-blind	Randomized-controlled	N/A

Outcomes

- **Primary outcomes:**

en
It is expected to identify the level of learning of students in the control and experimental groups regarding the teaching of Leadership, using an Online Module compared to a Traditional Expository Class, based on the observation of a variation of at least 10% in pre- and post-intervention, used to verify whether the outcome actually occurred

pt-br
Espera-se identificar o nível de aprendizagem dos discentes do grupo controle e experimental acerca do ensino de Liderança, utilizando-se um Módulo Online comparado a uma Aula Tradicional Expositiva, a partir da constatação de uma variação de pelo menos 10% nas medições pré e pós-intervenção, utilizados para verificar se o desfecho realmente ocorreu

- **Secondary outcomes:**

en
No secondary outcomes are expected

pt-br
Não são esperados desfechos secundários

Contacts

- **Public contact**
  - **Full name:** Daniele Vieira Dantas
  -
    - **Address:** Rua Petra Kelly, 61
    - **City:** Parnamirim / Brazil
    - **Zip code:** 59152-330
  - **Phone:** +5584999367260
  - **Email:** daniele00@hotmail.com
  - **Affiliation:** Universidade Federal do Rio Grande do Norte
- **Scientific contact**
  - **Full name:** Daniele Vieira Dantas
  -
    - **Address:** Rua Petra Kelly, 61
    - **City:** Parnamirim / Brazil
    - **Zip code:** 59152-330
  - **Phone:** +5584999367260
  - **Email:** daniele00@hotmail.com
  - **Affiliation:** Universidade Federal do Rio Grande do Norte
- **Site contact**
  - **Full name:** Daniele Vieira Dantas
  -
    - **Address:** Rua Petra Kelly, 61
    - **City:** Parnamirim / Brazil
    - **Zip code:** 59152-330
  - **Phone:** +5584999367260
  - **Email:** daniele00@hotmail.com
  - **Affiliation:** Universidade Federal do Rio Grande do Norte

Additional links:

- Download in ICTRP format

Total de Ensaios Clínicos 14527.

Existem 7071 ensaios clínicos registrados.

Existem 4004 ensaios clínicos recrutando.

Existem 183 ensaios clínicos em análise.

Existem 5118 ensaios clínicos em rascunho.

cadastre um novo usuário

notícias

sobre

ajuda

contato

equipe

links úteis

glossário

Organização Pan-Americana da Saúde World Health Organization FIOCRUZ SUS MINISTÉRIO DA SAÚDE GOVERNO FEDERAL BRASIL UNIÃO E RECONSTRUÇÃO
