# Supplementary material for: Effect of the online module on leadership in knowledge acquisition among nursing students: A randomized controlled study protocol
Source: PLoS One. 2025 Mar 25;20(3):e0320208. doi: 10.1371/journal.pone.0320208 (PMC11936248; doi:10.1371/journal.pone.0320208)
Supplement: Attachment — Submitted filename: Comments.docx [file pone.0320208.s007.docx]

The authors have successfully addressed the reviewer's comments. However, the comment posed by the previous reviewer about the outcome measurement that how often will the authors measure the outcome parameters still seems to be unaddressed.

I have also included a few additional minor suggestions for further improvement below.

**Title Geographical location:** The title of the manuscript effectively conveys the focus of the research, however, to enhance clarity and specificity, it would be beneficial to include the geographical area where the research is going to be conducted. For example, " Effect of the Online Module on Leadership in Knowledge Acquisition among Nursing in Students in Nepal: A Randomized Controlled Study Protocol" would provide readers with a clearer understanding of the study's context (1,2)

**Data Analysis:** The plan mentions using the Content Validity Index (CVI) and the Kappa test to assess the normality of data distribution. However, these tests are not designed for normality assessment. The CVI is used to measure the validity of items, often in survey research, and the Kappa test measures inter-rater reliability. Instead, normality should be evaluated using tests like the Shapiro-Wilk or Kolmogorov-Smirnov test. This limitation may lead to an inaccurate assumption about data distribution, affecting the choice of statistical tests. (275)

While the authors designate this study as a randomized controlled trial (RCT), there is frequent reference to it as a clinical trial in the abstract. (27,36) To avoid potential confusion, it may be helpful to consistently use the term “randomized controlled trial” to highlight the study’s design rigor and emphasize the randomization process, which is a critical feature in distinguishing it from other types of clinical trials.
