# Supplementary material for: Effect of the online module on leadership in knowledge acquisition among nursing students: A randomized controlled study protocol
Source: PLoS One. 2025 Mar 25;20(3):e0320208. doi: 10.1371/journal.pone.0320208 (PMC11936248; doi:10.1371/journal.pone.0320208)
Supplement: Attachment — Submitted filename: Response to Reviewers.docx [file pone.0320208.s008.docx]

**Response to Reviewers**

Dear Editor and Reviewers,

Thank you for the suggestions made in the manuscript. We are happy to know that the manuscript was reviewed with great care and attention. We make changes according to the requests of each reviewer and editor. We are available for further clarification. The changes were highlighted in the text of the manuscript in red. Furthermore, the answers in the response letter are in red. Follow the changes made:

- The title clinical trial is not appropriate, the setting of the trial is at school and not a clinical site or not involving patients or client- Unless there is some information I couldn’t access in the manuscript.

**Response:** Effect of the online module on Leadership on the knowledge acquisition of nursing students: protocol of a randomized controlled trial

- Sample size: The sample size is not clear (140,141). Kindly restate it clearly.

**Response**: The sample will be for convenience and will include only one class, composed of an average of 40 participants, with the participants randomly divided between the control group and the experimental group..

- Recruitment / Sampling strategy: the name of the randomised technique should be indicated ie. simple, systematic….? (122)
  - The recruitment and allocation is not so clear and it inconsistent under each section it appeared (153-157&172 to 175). Restructure and ensure it flows for easy comprehension.
  - With regards to the statement “interested students must register using a form Google forms” (153). what are the students registering for? course, to participate in the study? The students should also register the course in the SIGAA before recruitment into the study (150, 175 &187). clarification of these registrations is important.
  - The point at which participants are to be recruited and when they will sign the consent form is inconsistent across subsections (170 and 181) . Also how will the authors divide the two study groups? what strategy will they adopt (156)

**Response**: The recruitment of participants will be conducted in an organized and transparent manner. First, an invitation will be extended to students in the sixth semester of the Nursing undergraduate program at the Federal University of Rio Grande do Norte through the class's WhatsApp group. This invitation will encourage them to enroll in an extension course on leadership.

Interested students must fill out a registration form provided via the Google Forms platform. This form will collect initial participant information and confirm their interest in joining the study. Regarding consent, participants will sign the Informed Consent Form (ICF) at the time of course registration, that is, before the start of activities and before recruitment for the study.

After registration, a Google Meet call will be scheduled with all the registered students. During this call, a pre-test will be administered to assess the participants' prior knowledge on the topic of leadership. Recruitment will be completed with the confirmation of participation from students who sign the consent form and complete the pre-test. This process will ensure that all participants are properly informed and have given their consent to take part in the study.

To ensure random allocation to the groups, simple randomization will be applied using the website www.random.org. This tool will perform the drawing completely randomly, without interference from the researcher, ensuring that all participants have an equal chance of being assigned to the control or experimental group.

Participants in the control group will attend a traditional lecture, held in person at the Department of Nursing. The class, taught by the researcher, will last four hours in the afternoon and cover the same topics that will be addressed in the online educational module. Although the lecture itself is only four hours long, it will be part of a course that, in total, will be considered as having 30 hours, since it includes the time from registration to the completion of activities, for both groups. After the lecture, control group participants will take the post-test.

Meanwhile, participants in the experimental group will take part in an online educational module with recorded lessons hosted on the Google Classroom virtual learning environment. Participants will have a five-day period to access and complete the module content, with a total workload of 30 hours, from registration to completion. The time of access to the platform will take into account the students' enrollment in the platform until the post-test is completed.

It is important to emphasize that the allocation of participants will be conducted in a single-blind manner, thus ensuring the impartiality of the process. Additionally, the assignment of participants will be kept confidential to avoid any external interference or bias on the part of the researcher or others.

- Duration of intervention: The duration for the intervention is not clearly stated, if the 4 hours is the duration for the intervention as indication in line 200 and 206.Will the intervention take place for only four hours or will it be four hours per week?, or will the it cover for the whole semester ? . what about the workload of 30 hour assigned to the course (150), Authors should reconcile this for clarity?

**Response:** The sample will consist of approximately 40 students who are part of the sixth semester and are regularly enrolled in the first semester of 2024. Throughout their participation, both online and remotely, participants will exclusively use their own mobile phones and internet access to complete the registration and pre-test.

The pre-test will be administered via Google Forms, and no prior support material will be provided. Participants will have 20 minutes to complete the pre-test, and the researcher will use a Google Meet room as a virtual space to host all participants, as everyone must complete the pre-test synchronously within the 20-minute time limit. The virtual platform's chat will be used to signal when participants have finished. All participants must complete the test within the estimated time.

Randomization will be conducted only with participants who complete the pre-test and meet the eligibility criteria. These students will be randomized into two groups: the control group and the experimental group. After randomization, participants will be informed via WhatsApp and email which group they belong to—whether they will attend the course in person at the Department of Nursing or participate in the online educational module. At no point will it be disclosed to the participants which group they belong to.

Participants in the experimental group will be enrolled in a Google Classroom course by the researcher, and instructions will be sent via email and WhatsApp. The online educational module, composed of interactive recorded lessons, will have a four-hour duration, supplemented by additional content such as digital materials, quizzes, and forums. Participants will have flexibility in terms of accessing the lessons, with no geographical limitations.

The control group will participate in an expository-dialogued lecture, held in person at the Department of Nursing at the Federal University of Rio Grande do Norte. Visual materials, such as PowerPoint presentations, will be used during the lecture. The topics covered will be the same as those addressed in the online module, with the same depth and scope.

The experimental group will need to attend the Department of Nursing at UFRN in person. After the control group's activity is completed, experimental group participants will also be present. Following this, the post-test will be initiated for both groups, conducted online through Google Forms. Participants will use their own electronic devices, accessing UFRN's internet. They will be given 20 minutes to complete the post-test. After its completion, all participants will receive a course participation certificate and be dismissed.

Therefore, the pre-test will be conducted synchronously and remotely, using Google Forms, with participants gathered in a Google Meet room. After the pre-test is completed, participants will be allocated and randomized into groups. The post-test will be conducted after all scheduled activities are completed, and this will be done in person, using Google Forms.

The aim of the evaluation is to assess the effectiveness of an online educational module on leadership in the knowledge acquisition of Nursing students.

- Intervention: You need to indicate how you will deal with confounding factors. The allocation of the intervention to the two treatment groups should be well defined from the beginning of the methodology, so that readers can know what exactly you are comparing.The proposal should show the differences between the group. ie CG=face to face instruction with traditional time of 4 hours…..? whiles EG= online teaching with flexibility of 5days with added component to the subject matter or teaching methodology such as clinical cases? it is also not clear the mode of teaching researchers will employ for the online class, will it be real-time or recorded lecture? (206 - 208). Please indicate.

**Response:** Participants in the experimental group will be enrolled in a Google Classroom course by the researcher, and the instructions will be sent via email and WhatsApp. The online educational module, consisting of interactive recorded lessons, will have a total duration of four hours, supplemented by additional content such as digital materials, quizzes, and forums. The topics covered include team motivation and engagement, organizational culture, conflict management, and constructive feedback. Participants will have flexibility regarding the timing of the lessons and will not face any geographical limitations.

The control group will participate in an expository-dialogued lecture, held in person at the Department of Nursing at the Federal University of Rio Grande do Norte. Visual materials, such as PowerPoint presentations, will be used during the lecture. The topics covered will be the same as those addressed in the online module, with the same depth and scope.

- Outcome measurement: How often will the authors measure the outcome parameters? is it after each lesson? This is not clear in the manuscript (267,272), What is to be measured for primary outcome is not clear as well as the tool they will use to measure it? (268-270).

**Response:** To evaluate the effectiveness of the module and knowledge acquisition, a data collection tool will be used in the form of a Google Forms questionnaire, which will serve as both the pre-test and post-test. This questionnaire is divided into three parts: a demographic characterization section; a section with a problem-based scenario composed of four objective questions aimed at assessing leadership-related topics such as team motivation and engagement, organizational culture, conflict management, and constructive feedback; and a third section featuring the adapted version of the Student Nurse Leadership Self-Perception Questionnaire (QUAPEEL) by Cardoso, Ramos, and D'Innocenzo (2014). The QUAPEEL consists of questions designed to explore students' knowledge of leadership, allowing for both qualitative and quantitative assessment. It also addresses the skills and attitudes that students believe they possess or need to develop.

Yours sincerely,

The authors.
