# Supplementary material for: Effect of the online module on leadership in knowledge acquisition among nursing students: A randomized controlled study protocol
Source: PLoS One. 2025 Mar 25;20(3):e0320208. doi: 10.1371/journal.pone.0320208 (PMC11936248; doi:10.1371/journal.pone.0320208)
Supplement: Attachment — Submitted filename: Response_to_Reviewers_auresp_2.docx [file pone.0320208.s009.docx]

**RESPONSE TO REVIEWERS**

Natal, January 20, 2025

Dear reviewers and editor of PLOS ONE,

As requested, the authors of the manuscript entitled " **Effect of the Online Module on Leadership in Knowledge Acquisition among Nursing Students: A Randomized Controlled Study Protocol**" (PONE-D-24-20769R1), have adapted the text respecting the recommendations given by the reviewers.

We would like to point out that a manuscript with the changes highlighted in YELLOW was sent to the journal for consideration and another manuscript was sent in a clean version.

The following changes were made:

**Text:** **As requested by the reviewers.**

**Reviewer 3**

- We would like to clarify that the use of the future tense in the text of the article is due to the fact that the protocol is still under development and preparation for the start of recruitment.

**Reviewer 10**

- We have deleted the words 'therefore' in lines 84 and 86 of the previous version of the article;
- **Page 8, line 183; Page 9, line 215 -** We have identified the mistake in the use of the terms 'online and remotely' and have adjusted them to 'online and in person', as suggested;
- **Page 12, lines 292-297 -** We have clarified in more detail where the data will be available after the research is completed.

**Title: As requested by the reviewers.**

**Reviewer 5**

- Regarding **reviewer 5's** suggestion to include the geographical area in the title of the study, we recognize the relevance of this recommendation. However, we have opted to keep the title as it is, as it is already long and, considering the word limitation for titles, we prefer to preserve it in its original form.

**Abstract: As requested by the reviewers.**

**Reviewer 8**

- **Page 2, lines 32-37 -** The abstract was revised to make it more concise, without duplication, and with a clear statement of the purpose of the study. In addition, the expected results of the research were explicitly included, as suggested.

**Reviewers 5, 9, 10 and 11**

- **Page 1, lines 26-27 -** We have revised the use of the term 'clinical trial' in the abstract and throughout the text of the article, replacing it with 'randomized controlled trial', as suggested.

**Reviewers 10 and 11**

- We identified that the abstract was duplicated in the text and only one version was kept;

**Method: As requested by the reviewers.**

**Reviewer 5**

- **Pages 13-14, lines 316-318 -** We identified a mistake in the use of the 'Content Validity Index' and the Kappa test. We have therefore adjusted the text, replacing these statistical tests with the Shapiro-Wilk test or the Kolmogorov-Smirnov test for assessing normality.

**Reviewer 7**

- **Page 5, lines 123-125 -** We have included a more detailed explanation of registration on Google Forms and SIGAA.
- **Page 6, lines 137-140 -** In addition, we have clarified how researchers will minimize the risk of knowledge contamination between groups.
- **Page 5, lines 109-114 -** In response to the request of **reviewer 7,** we have justified the choice of convenience sampling, highlighting the possible limitations associated with this type of sampling;
- **Page 9, lines 212- 214 -** We specified the frequency of the pre-test and post-test in the text.

**Reviewer 8**

- **Pages 9-11, lines 223-261** - In response to the reviewer's comments, we have clarified the data collection instrument in more detail. In the **“Data Collection Instrument”** section, we have added all the variables that will be assessed in the instrument for both groups. This ensures that the evaluation of all participants is carried out equally and without any bias.

**Reviewer 9**

- **Line 27; line 105; line 118; line 123; line 182 -** We identified a mistake regarding the semester of the students, clarifying that these will be 'sixth semester' students. All errors relating to the use of the terms 'period' and 'seventh semester' have been corrected in the text.

**Reviewer 10**

- **Page 7, lines 167-169 -** We have clarified in more detail the sample size and the restriction of equal sizes between the groups, as requested;
- **Pages 6-7, lines 152-158; lines 162-165** - In response to the reviewer's question, we provided a more detailed explanation of the course load for the intervention condition in the groups;
- **Page 9, lines 212-214 and lines 215-218** - In response to the request, we have added information on the duration of the online meeting for participants during the pre-test and the face-to-face meeting for the post-test;
- **Page 10, lines 271-281 -** We have clarified the type of blinding used in this research protocol;
- **Page 11, lines 226-261 -** We have provided more details on the study variables that will be collected;
- **Page 11, lines 271-276** - As suggested by the reviewer, we made the differences between in person and online modalities clearer during the consent process.

**Reviewer 11**

- **Page 12, lines 326-333** - We have more clearly specified the statistical tests that will be used to evaluate the effect of the intervention between the groups.
- **Page 5, lines 109-114** - In response to **reviewer 11's** comment, we have clarified the sample size and generalizability of the study in more detail;
- **Page 6, lines 147-149** - In response to the reviewer's comment, we have clarified the process of randomizing the students.

**Results: As requested by the reviewers.**

**Reviewer 8**

- **Page 14, lines 345-248 -** We justified the absence of results by explaining that this is a research protocol and therefore only the expected results are available.
- **Lines 350-356 -** In addition, we provide more details on the primary results of the study.

**Discussion: As requested by the reviewers.**

**Reviewer 10**

- **Pages 15-17, lines 368-399 -** In response to **reviewer 10**, we have clarified in the discussion of this protocol the differences between in person and online modalities of the leadership course, using references from authors who deal with this subject.

**Conclusion: As requested by the reviewers.**

**Reviewer 8**

- We respectfully justify that it is not possible to include a conclusion section summarizing the findings of the study, as the research has not yet started and therefore there are no final results to be presented.

**References: As requested by the reviewers.**

**Reviewer 10**

- We revised all the references, translating them into English. In addition, we adjusted the references to Vancouver format, in accordance with PLOS ONE guidelines.

We would like to thank the reviewers for their corrections and for the opportunity to review our article for consideration and possible acceptance by the renowned PLOS ONE journal.

Yours sincerely,

The authors
